# Lactoferrin-derived peptide PXL01 impacts nerve regeneration after sciatic nerve reconstruction in healthy and diabetic rats

**DOI:** 10.3389/fcell.2025.1565285

**Published:** 2025-04-07

**Authors:** Derya Burcu Hazer Rosberg, Margit Mahlapuu, Raquel Perez, Lars B. Dahlin

**Affiliations:** ^1^ Department of Translational Medicine – Hand Surgery, Lund University, Malmö, Sweden; ^2^ Department of Hand Surgery, Skåne University Hospital, Malmö, Sweden; ^3^ Department of Chemistry and Molecular Biology, Göteborg University, Göteborg, Sweden; ^4^ Unit for Social Epidemiology, Department of Clinical Sciences (Malmö), Lund University, Malmö, Sweden; ^5^ Department of Biomedical & Clinical Sciences, Linköping University, Linköping, Sweden

**Keywords:** PXL01, nerve reconstruction, autograft, nerve regeneration, diabetes, Goto-Kakizaki rat, axonal outgrowth

## Abstract

**Introduction:**

Although advanced surgical techniques are available, satisfactory functional outcomes after peripheral nerve injuries are uncommon. Hence, immune-modulating factors such as PXL01, a lactoferrin-derived peptide that improves axonal outgrowth in injured human digital nerves, have gained attention. We previously reported a short-term immunosuppressive effect of PXL01 after the repair of transected rat sciatic nerves, but it had no effect on nerve regeneration. Here, we investigated the potential of PXL01 to improve nerve regeneration in healthy rats and in a rat model of type 2 diabetes (Goto-Kakizaki [GK] rats).

**Methods:**

A 10-mm sciatic nerve defect was created in healthy (n = 14) and diabetic GK rats (n = 14) and reconstructed using nerve autografts. Immediately after surgery, PXL01 or sodium chloride (control, placebo) (n = 7 for each treatment) was administered around the autograft. On day 8, immunohistochemical staining of the sciatic nerve and dorsal root ganglia (DRGs) was performed to analyze axonal outgrowth (neurofilament staining); inflammation (CD68 and CD206 macrophage staining in nerve); Schwann cell and sensory neuron activation (transcription factor ATF3 staining in nerve and DRGs) and apoptosis (cleaved caspase 3 staining in nerve); and neuroprotection (heat shock protein [HSP27] staining in nerve and DRGs).

**Results:**

PXL01 had no impact on the macrophage response in the autografts but increased axonal outgrowth and HSP27 expression in the DRGs of healthy and diabetic rats, despite a lower number of activated Schwann cells in the autograft. Diabetes affected axonal outgrowth, Schwann cell and macrophage responses, and HSP27 expression. These effects were observed in the sciatic nerve as well as the DRG.

**Discussion:**

Application of PXL01, despite having no impact on macrophages, may improve axonal outgrowth and affects Schwann cell activation in autograft-reconstructed sciatic nerves, as well as conveys neuroprotection (HSP27 expression) in the DRGs of healthy and diabetic GK rats. Diabetes influenced nerve regeneration in such autografts. Therefore, PXL01 is a promising candidate to improve nerve regeneration.

## 1 Introduction

Transection or laceration injury of the nerve trunk requires surgical intervention, such as primary nerve repair or reconstruction using autologous nerve grafts (Dahlin, 2008; Dahlin, 2013; Pan et al., 2020). Despite advancements in microsurgical techniques, functional outcomes are still suboptimal for a variety of reasons, including the complexity of cellular interactions in the degeneration and regeneration processes (Dahlin, 2023; Lanier et al., 2021; Lin and Jain, 2023). There is still a lack of detailed knowledge about these cellular interactions and how they can be influenced or stimulated, especially in individuals with diabetes. Many factors affect the microenvironment of the nerve and directly influence neurons, which may impact nerve regeneration upon injury or cause the development of diabetic neuropathy (Cattin and Lloyd, 2016; Dahlin, 2023). In addition to adequate timing of surgery and application of appropriate surgical techniques, pharmacological treatment to enhance nerve regeneration can be an effective strategy; however, few clinically approved agents are available (Bolandghamat and Behnam-Rassouli, 2020). The immunosuppressant tacrolimus (FK506) has been approved by the FDA for certain indications and is associated with improved nerve regeneration (Daeschler et al., 2023).

PXL01 is a synthetic peptide produced from lactoferrin (Mahlapuu et al., 2020). In experimental studies in rabbits using PXL01, a reduction in postsurgical adhesions after tendon surgery was observed (Hakansson et al., 2012; Wiig et al., 2011). Furthermore, recent clinical data indicates that PXL01 may act locally as an antiadhesive pharmacological agent after flexor tendon surgery in the human hand (Wiig et al., 2014). The antiadhesive mechanism of PXL01 is linked to increased expression of the *PRG4* gene, which encodes the proteoglycan lubricin (Edsfeldt et al., 2017). In addition, PXL01 has been shown to reduce the expression of plasminogen activator inhibitor-1 (PAI-1), which is an inhibitor of the plasminogen activator system, a pathway that may affect nerve regeneration (Siconolfi and Seeds, 2001). Specifically, PAI-1 inhibits Schwann cell migration from dorsal root ganglia (DRGs) (Nilsson et al., 2005), indicating that PXL01 may stimulate nerve regeneration. Signs of improved axonal outgrowth were observed consistently in transected human digital nerves in a clinical study on flexor tendon surgery (Wiig et al., 2014). However, these positive results were not supported by a recent experimental study of PXL01 in primary repair of transected rat sciatic nerves (Hazer Rosberg et al., 2024). Local application of PXL01 soon after repairing a nerve injury in healthy Wistar rats decreased the inflammatory response (indicated by CD68 staining of pan-macrophages) but did not affect pro-healing macrophages (CD206-stained macrophages), axonal outgrowth, or Schwann cell response (Hazer Rosberg et al., 2024). One hypothesis is that PXL01 may stimulate nerve regeneration after nerve reconstruction with an autologous nerve graft, which is initially avascular before angiogenesis has been initiated (Cattin et al., 2015). This has been shown not only in healthy rats, but also in diabetic Goto-Kakizaki (GK) rats, a model resembling type 2 diabetes (Dahlin, 2023; Stenberg et al., 2016; Stenberg et al., 2017). Therefore, our aim was to investigate the axonal outgrowth and responses of Schwann cells, sensory neurons, and macrophages after local application of PXL01 to an autograft reconstruction of the sciatic nerve in healthy Wistar and diabetic GK rats.

## 2 Materials and methods

### 2.1 Animals and surgery

Ethical approval was obtained from the Animal Ethics Committee of Lund University, Lund, Sweden (protocol code: 5.8.18–06842/2019).

Healthy female Wistar rats (n = 14) and diabetic GK (n = 14) rats (8 weeks old, body weight 200–220 g each) were randomly divided into two groups, as in previous designs (Hazer Rosberg et al., 2024; Stenberg and Dahlin, 2014; Stenberg et al., 2017). The treatment groups received either PXL01 in sodium hyaluronate (HA) (n = 7 for each rat strain) or sodium chloride alone (placebo; n = 7 for each rat strain). After inducing anesthesia with intraperitoneal injection of 1 mL/100 g (1 mg/kg) Ketalar (10 mg/mL; Pfizer, Helsinki, Finland) and 0.25 mL/100 g (0.5 mg/kg) Rompun (20 mg/mL; Bayer Healthcare, Leverkusen, Germany), the left sciatic nerve was exposed at the hind-limb level. A 10-mm segment of the sciatic nerve was transected using micro-scissors and rotated on its long axis. It was immediately reconstructed as an autograft by securing it with three single sutures of 9–0 Ethilon in the epi-/perineurium (Ethicon, Johnson and Johnson, Livingston, United Kingdom).

**FIGURE 1:**
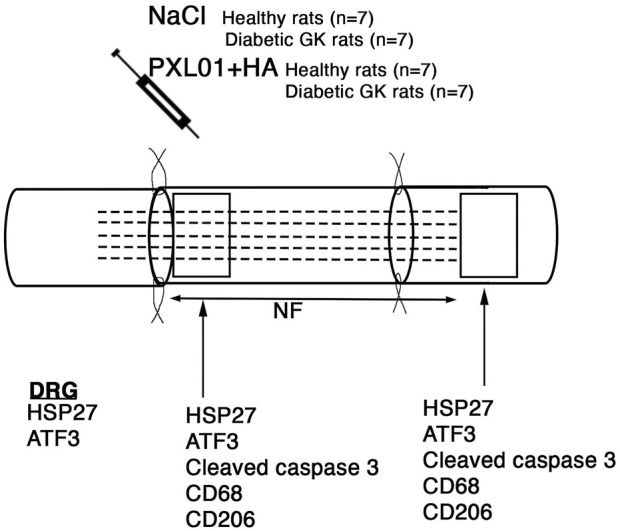
Schematic overview of the surgical method, the substances applied locally, and areas analyzed on the operated sciatic nerve. The presence of axonal outgrowth (length of neurofilament, NF; indicated by dashed lines), activated (ATF3-stained) and apoptotic (cleaved caspase 3-stained) Schwann cells, CD68 (activated, pan-macrophage)- and CD206 (pro-healing)-stained macrophages together with heat shock protein 27 (HSP27) expression at both the proximal lesion site (just distal to the proximal suture line) and at the distal nerve end (just distal to the distal suture line where there are no axons present at 8 days) were immunohistochemically analyzed at 8 days. In addition, immunoreactivity for HSP27 and ATF3 were analyzed in the bilateral dorsal root ganglia (DRGs).

Immediately after reconstruction, PXL01 or NaCl was applied locally to the reconstructed nerve (Figure 1), according to a previous study (Hazer Rosberg et al., 2024). In our previous study we used both NaCl and the vehicle HA together with the experimental drug PXL01 with the vehicle HA and analyzed nerve regeneration after primary repair of a nerve transection injury. Those analyses showed no significant differences with respect to axonal outgrowth (Hazer Rosberg et al., 2024). Therefore, and in view of the 3R principle for research on animals (reduction, refinement and replacement), we decided to only include two groups in the present study with application of NaCl or PXL01 in HA as pharmacological agents. The rats in the placebo-NaCl group (n = 7) received 0.2 mL NaCl solution on the reconstructed nerve, and those in the PXL01 group received 0.2 mL of PXL01 in HA (n = 7). After closing the layers, Temgesic 0.01–0.05 mg/kg (0.3 mg/mL; Schering-Plough Europe, Brussels, Belgium) was injected intraperitoneally for pain relief. The animals were followed for 8 days, which has been shown to be sufficient for axonal outgrowth and evaluation of the early regeneration process in autograft-reconstructed nerve injury models (Kerns et al., 1993). An overdose of pure pentobarbital sodium (Allfatal vet. Pentobarbital [100 mg/mL]; Omnidea AB, Stockholm, Sweden) and subsequent heart puncture were used to sacrifice the rats. The experimental and uninjured sciatic nerves and the corresponding DRGs (L4 and L5) were harvested bilaterally.

### 2.2 Pharmacological drug

PXL01 concentrate, a product of Promore Pharma AB (Fogdevreten 2, SE-171 65 Solna, Sweden), was applied as in our previous study (Hazer Rosberg et al., 2024). In the operating theater, 0.4 mL of 50 mg/mL PXL01 (20 mg in total) concentrate was mixed with 0.6 mL of 25 mg/mL HA (15 mg in total; as a carrier) to a final concentration of 20 mg/mL PXL01 and 15 mg/mL HA. PXL01 in HA (0.2 mL, 4 mg of PXL01; 3 mg HA) was directly applied to the surface of the reconstructed sciatic nerve. The rats in the placebo-treated group received 0.2 mL of NaCl under the same conditions.

### 2.3 Harvest of the specimens

The specimens were harvested and processed in a similar manner as in our previous studies (Hazer Rosberg et al., 2021; Hazer Rosberg et al., 2024; Stenberg et al., 2021). In brief, the harvested specimens were fixed in Stefanini’s solution (4% paraformaldehyde and 1.9% picric acid in 0.1 M phosphate buffer, pH 7.2) for 24 h, then washed in 0.01 M phosphate-buffered saline (PBS) and placed in 20% sucrose in 0.01 M PBS for 24 h. The specimens were then embedded in O.C.T. Compound (Histolab products AB, Gothenburg, Sweden) and frozen immediately in a cryostat. Longitudinal 8-μm thick sections were prepared, mounted on Super Frost plus slides (Thermo Scientific, Braunschweig, Germany), and stored at −20°C until processing.

### 2.4 Immunohistochemistry

The immunohistochemistry protocol was described in our previous studies (Hazer Rosberg et al., 2021; Hazer Rosberg et al., 2024; Stenberg et al., 2021). Briefly, after the washing procedure, the slides were incubated with primary antibodies in 0.25% Triton X-100% and 0.25% bovine serum albumin in PBS overnight at 4°C. The primary antibodies used are listed in Supplementary Table S1. Immunohistochemical analysis was performed to detect axonal outgrowth (neurofilament [NF] expression); neuroprotection in the sciatic nerves and DRGs (heat shock protein 27 [HSP27] expression); sensory neuron activation (activating transcription factor 3 [ATF3] expression); and activation and apoptosis in Schwann cells in the sciatic nerve (expression of ATF3 and cleaved caspase 3, respectively). Macrophages were also analyzed in accordance with our previous study using anti-CD68 (ED1) as pan-macrophage marker and anti-mannose receptor (CD206) antibody to detect pro-healing (M2 type) macrophages. The next day, the sections were rinsed with PBS and incubated in secondary antibody solutions for 1 h at room temperature in the dark. The list of secondary antibodies is presented in Supplementary Table S1, and they are the same as those used in our previous study (Hazer Rosberg et al., 2024). After incubation, the slides were washed with PBS (3 × 5 min), mounted with Vectashield Mounting Medium with DAPI (4′,6-diamino-2-phenylindole) (Vector Laboratories, Burlingame, CA, United States), and cover-slipped. The validity of all primary antibodies was checked using negative controls in both experimental and contralateral sciatic nerve tissues and in tissues from naïve Wistar and GK rats (Hewitt et al., 2014; Stenberg et al., 2021).

As in our previous studies, double staining was also performed on sciatic nerve tissues with S-100 and NF antibodies to verify that ATF3- and cleaved caspase 3-stained cells were Schwann cells. HSP27 expression and the localization of CD68- and CD206-stained macrophages in the sciatic nerve were also verified with respect to regenerating axons and Schwann cells [data not shown; for detailed information, please see (Hazer Rosberg et al., 2024)].

### 2.5 Imaging and analysis

A digital camera (Olympus DP80; Tokyo, Japan) attached to a motorized reflected fluorescence microscope (Olympus BX3; Tokyo, Japan) was used to image the sections. Analysis was performed using Cell Sens Dimension software (Olympus, Tokyo, Japan). Imaging and analysis of the operated sciatic nerve, contralateral unoperated sciatic nerve, and DRGs were performed as described previously (Hazer Rosberg et al., 2024). Sciatic nerve sections were analyzed on day 8 after treatment at two different sites, as previously described: at the proximal side of the lesion (distal to the proximal suture line) and the distal side of the lesion (distal to the distal suture line, where the regenerating axons have not yet reached at 8 days) (Figure 1) (Hazer Rosberg et al., 2021; Stenberg et al., 2016).

The images of ATF3, cleaved caspase 3, CD68, and CD206 staining in the sciatic nerve were analyzed under 20× magnification, and the HSP27-stained images of the sciatic nerve were analyzed under 10× magnification. The DRG images and NF immunostaining in the sciatic nerve was performed using the live process function in Cell Sens Dimension. The length of axonal outgrowth (stained NF proteins) was determined from the starting point at the proximal suture line to the end point of the longest growing axons, according to a previously described method (Hazer Rosberg et al., 2021; Stenberg et al., 2021; Stenberg et al., 2016). All immunohistochemical analyses were analyzed by a researcher blinded to the treatment modality using three random slides from each rat, and the mean value was calculated.

### 2.6 Immunohistochemical analysis

ImageJ 1.53a (National Institutes of Health, Bethesda, MD, United States, http://imagej.nih.gov/, accessed on 03 June 2022) was used to analyze HSP27 expression in the operated and contralateral uninjured sciatic nerves and DRGs. We previously described in detail the analysis method using the ImageJ software and reported the expression of HSP27 in both axons and Schwann cells in HSP27/NF and HSP27/S-100 double staining experiments (Stenberg et al., 2021). The data for HSP27 immunoreactivity in the sciatic nerve and DRG are expressed as a percentage of immunoreactivity per total selected area. Data from the L4 and L5 DRGs from the experimental and contralateral sides were pooled for each rat and are presented as experimental or contralateral L4-L5 DRG. To describe the change in immunoreactivity on the experimental nerve side relative to the contralateral non-operated side, HSP27 expression is presented as the ratio of the experimental side at the specific lesion site to the contralateral side. Similarly, HSP27 expression in the DRGs is presented as the ratio of HSP27 expression in the experimental DRG to that in the contralateral DRG.

ATF3- and cleaved caspase 3-stained Schwann cells were counted manually (20× magnification) both at the proximal side of the lesion (just distal to the proximal suture site) and at the distal nerve end, i.e., the area just distal to the distal suture line, where the regenerating axons have not yet reached at 8 days. The number of ATF3- and cleaved caspase 3-stained Schwann cells was expressed as a percentage of the total number of DAPI-stained cells that were quantified using ImageJ. The total number of DAPI-stained cells was quantified using a previously described plug-in (Woeffler-Maucler et al., 2014) for ImageJ (Hazer Rosberg et al., 2021; Hazer Rosberg et al., 2024). Data are expressed as mean values from three random slides. In whole DRG images, the number of ATF3-stained sensory neurons is expressed as a percentage of the total number of sensory neurons (counted manually). The mean values of the data gathered from the DRGs are presented as percentages.

As in our previous study (Hazer Rosberg et al., 2024), we used the CD68 antibody to identify the total number of “activated” pan-macrophages (Jia et al., 2019; Ydens et al., 2020; Zigmond and Echevarria, 2019), and CD206 was selected as a marker to visualize anti-inflammatory/pro-healing (M2) macrophages (Liu et al., 2019; Mokarram et al., 2012). CD68- and CD206-stained macrophages were counted manually in sections at both the proximal side of lesion and distal nerve end. The data are presented as the percentage of activated pan-macrophage (CD68) count or the pro-healing macrophage (CD206) count to the total number of DAPI-stained cells quantified with ImageJ. Data are presented as the mean values of the macrophage counts from three slides. Additionally, the contralateral sciatic nerve showed staining of the M2 macrophages with CD206; therefore, they were included in the presented data (Hazer Rosberg et al., 2024). The amount of CD206-stained macrophages is expressed as a ratio (experimental side/control side).

### 2.7 Statistical methods

Data are expressed as median values with the 25th and 75th percentiles (interquartile range). The nonparametric Kruskal–Wallis (KW) test and Mann–Whitney U-test were used as *post hoc* tests to analyze any significant differences between all treatment groups. To determine any significant differences between the proximal and distal nerve areas of the sections within the same treatment groups, and the difference between the contralateral unoperated side and the experimental side, the nonparametric Wilcoxon signed rank test was used. Spearman’s correlation test was used (r > 0.30 considered relevant) to analyze any correlation between the immunohistochemical data. Two-way ANOVA was conducted to examine the effect of the type of rat and treatment on axonal outgrowth, Schwann cell response in the sciatic nerve, macrophage response, neuroprotective effect based on HSP27 expression in the nerve or DRG, and sensory neuronal response in DRGs. The used classification (0.01–0.059 = small, 0.06–0.139 = medium, 0.14 and above = large) was an adaptation of the guidelines proposed by Cohen (1988) for effect size in statistical tests. Statistical analyses were performed using SPSS Statistics software (version 28; IBM, Armonk, NY, United States) or Stata v14.1 (StataCorp, College Station, TX, United States) with a p-value <0.05 considered significant.

## 3 Results

### 3.1 Axonal outgrowth

**FIGURE 2:**
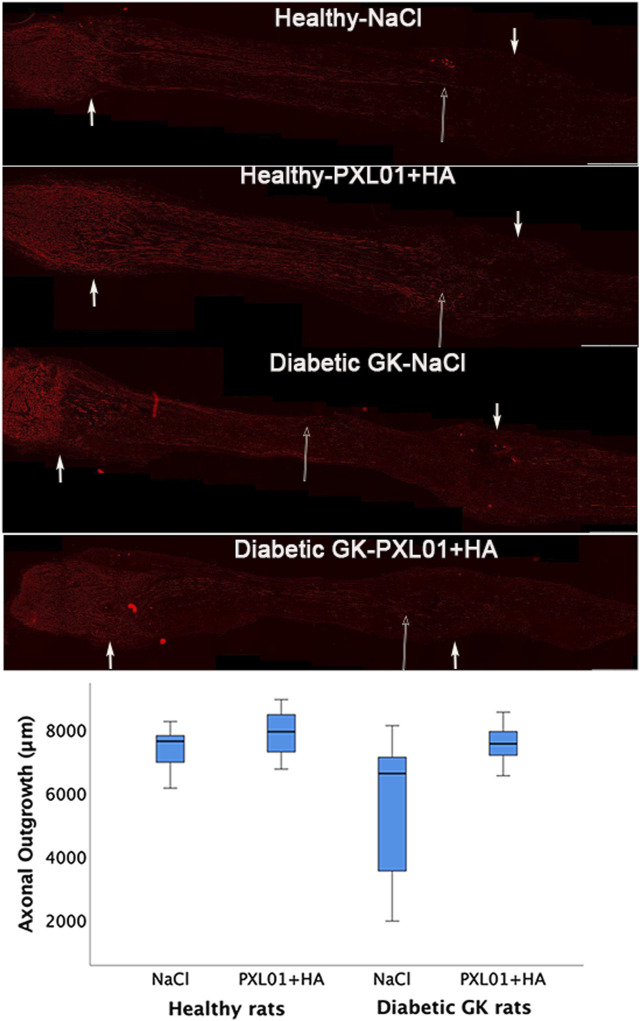
Axonal outgrowth in nerve autografts 8 days after nerve reconstruction in healthy and diabetic Goto-Kakizaki (GK) rats with different treatments applied locally with sodium chloride (NaCl group) or PXL01 with sodium hyaluronate carrier (PXL01+HA group) is indicated by neurofilament staining. Scale bar = 1 mm. Small arrows in both ends show the suture sites. Long arrow shows the longest neurofilament staining. Box plots indicate the 25th and 75th percentiles (Tukey’s hinges) with the horizontal line in the middle indicating the median value. Error bars show minimum and maximum values.

Axonal outgrowth was determined by measuring the longest axonal NF protein immunoreactivity at the proximal suture line of the reconstructed sciatic nerve. It was detected in all treatment groups at approximately 7,500 μm, with the highest median values in the PXL01-treated group in healthy rats and the lowest median values in the diabetic GK rats, but with no overall statistically significant differences in the treatment groups, as evaluated by KW analysis (p = 0.08; Figure 2; Table 1).

**TABLE 1:** Immunohistochemical analysis of the regeneration process in the sciatic nerve, using different antibodies, at 8 days in the autograft reconstructed sciatic nerve injury model in healthy Wistar rats and diabetic Goto-Kakizaki (GK) rats after treatment with sodium chloride (NaCl), and PLX01 in sodium hyaluronate (PXL01+HA).

Sciatic nerve		Healthy Wistar rats		Diabetic Goto-kakizaki rats		
		NaCl (n = 7)	PXL01+HA (n = 7)	NaCl (n = 7)	PXL01+HA (n = 7)	p-values
Length of axonal outgrowth (neurofilament proteins; µm)		7,612 (6,944–7,874)	7,914 (6,839–8,768)	6,599 (2,894–7,442)	7,534 (7,095–7,998)	0.08
HSP27 - contralateral side (% total area)		7.7 (6.3–9.0)	7.8 (7.3–9.3)	9.6 (9.0–10.5)	8.6 (8.1–9.5)	0.12
HSP27 - experimental side (% total area)	Site of lesion	17.4 (11.6–22.5)	15.6 (11.2–20.3)	16.1 (15.7–17.7)	14.9 (12.8–17.7)	0.68
	Distal nerve end	13.5 (12.1–21.9)	12.5 (11.4–13.3)	12.2 (11.1–16.6)	15.7 (9–19.9)	0.65
HSP27 ratio - experimental/contralateral	Site of lesion	2.1 (1.6–2.8)	2.1 (1.3–2.6)	1.7 (1.4–2.3)	1.8 (1.4–1.9)	0.40
	Distal nerve end	1.7 (1.4–3.9)	1.6 (1.4–2.0)	1.6 (1.0–1.7)	1.9 (1.1–2.4)	0.62
ATF3 - experimental side (% total number DAPI positive cells)	Site of lesion	10.0 (6.1–11.8)^a,b^	8.3 (5.7–8.6)^b^	3.4 (2.0–7.3)	1.8 (1.5–2.3)	**0.001**
	Distal nerve end	13.3 (10.0–21.7)^a,b^	18.9 (14.6–22.5)^a,b^	5.2 (1.3–7.4)	3.0 (1.5–6.5)	**<0.001**
Cleaved caspase 3 - experimental side (% total number DAPI positive cells)	Site of lesion	8.7 (8.4–11.7)	8.9 (7–9.9)	9.3 (8.6–13.8)	9.4 (8.8–11.5)	0.67
	Distal nerve end	10.4 (9.6–11.4)	11.6 (9.9–14.7)	11.4 (10.7–12.5)	11.1 (9.0–14.3)	0.42

Axonal outgrowth is represented by the length of neurofilament (NF) staining (µm). HSP27 immunoreactivity is presented as a percentage of the total area (%). The number of ATF3- and cleaved caspase 3-stained Schwann cells is expressed as a percentage of the total number of DAPI-positive cells. Data are presented as median (25th–75th percentiles). P-values were based on the Kruskal–Wallis test. Significant p values (p < 0.05) are indicated in bold. DAPI, 4′,6-diamino-2-phenylindole. NaCl: sodium chloride treatment group, PXL01+HA: PXL01 in sodium hyaluronate carrier treatment group.

^a^ indicates significant difference compared with the GK-NaCl group.

^b^ indicates significant difference compared with the GK-PXL01 group.

### 3.2 HSP27 immunoreactivity in sciatic nerve

**FIGURE 3:**
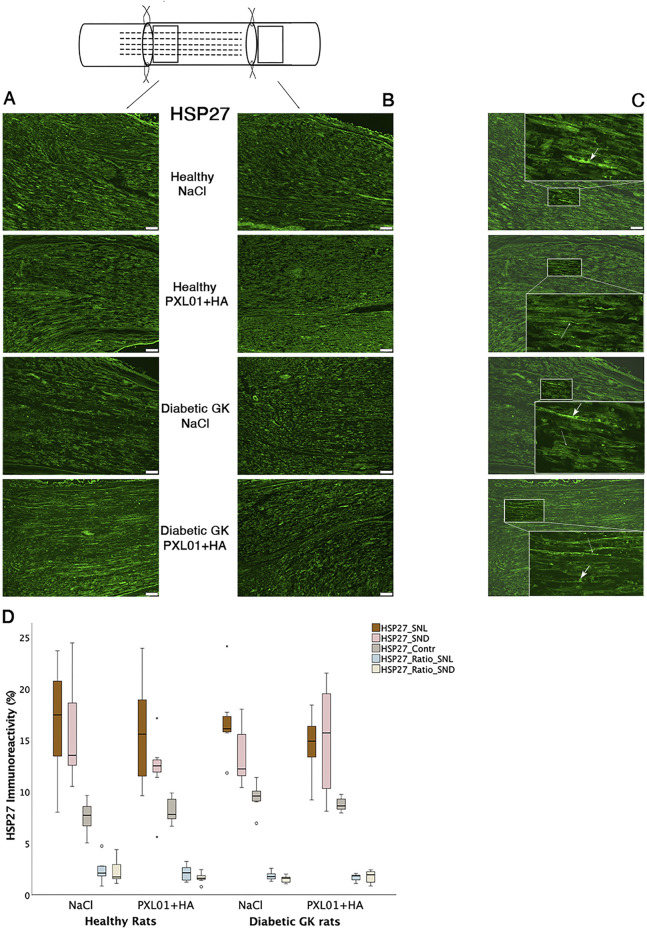
HSP27 immunoreactivity at the proximal side of lesion **(A)** and at the distal nerve end **(B)** in autograft reconstructed sciatic nerves 8 days after the application of different treatment agents in Wistar and in diabetic GK rats. Scale bar = 100 μm. Box plots **(D)** show the HSP27 immunoreactivity in the operated sciatic nerve at the proximal side of lesion (SNL) and at distal distal nerve end (SND) as well as in the contralateral nerves (Cont). The groups are coded according to different treatment agents: sodium chloride treated group, NaCl; and PXL01 with sodium hyaluronate treated group, PXL01+HA. The images indicating the proximal site of lesion in SNL in all four experimental groups have a magnified insert indicating the Schwann cell expressing the HSP27 designated with short arrow, and the axonal expression of HSP27 with long arrow **(C)**. Box plots indicate the 25th and 75th percentiles (Tukey’s hinges) with the horizontal line in the middle indicating the median value. Error bars show minimum and maximum values.

HSP27 expression was detected in Schwann cells and outgrowing axons, as confirmed by double-staining for S-100 and NF, respectively. It was analyzed in the contralateral uninjured nerve and operated sciatic nerve, both at the proximal side of the lesion (just distal to the proximal suture line) and at the distal nerve end (beyond the autograft) (Figure 1). Expression of HSP27 was significantly higher in the experimental groups both at the proximal side of the lesion (p = 0.02 for the Wistar-NaCl group, p = 0.02 for the Wistar-PXL01 group; p = 0.02 for the GK-NaCl group, p = 0.02 for the GK-PXL01 group; Wilcoxon signed rank test) and at the distal nerve end than that of the contralateral side for each treatment group (p = 0.02 for the Wistar-NaCl group, p = 0.04 for the Wistar-PXL01 group, p = 0.02 for the GK-NaCl group, p = 0.04 for the GK-PXL01 group; Wilcoxon signed rank test). HSP27 expression in the operated sciatic nerve and the ratio of HSP27-expressing cells in the operated sciatic nerve to that in the contralateral nerve showed similar results in all treatment groups, with no statistical significance between the treatment groups (p = 0.68 at site of lesion, p = 0.65 at the distal nerve end, and p = 0.40 ratio at site of lesion, p = 0.62 ratio at the distal nerve end; KW test; Figure 3; Table 1). We detected no statistically significant difference between the proximal side of the lesion and the distal nerve end in each treatment group (p = 0.73 for the Wistar-NaCl group, p = 0.18 for the Wistar-PXL01 group; p = 0.12 for the GK-NaCl group; p = 0.87 for the GK-PXL01 group; Wilcoxon signed rank test).

In our previous study with local application of PXL01 on a primary nerve repair (Hazer Rosberg et al., 2024), we also conducted a pilot study including single naïve and sham operated rats with NaCl, HA and PXL01 application followed by similar analysis as in the present project (see Supplementary Material in Hazer Rosberg et al. (2024)) and revealing no obvious differences between naïve, sham-operated (both sham-operated side and contralateral sides) and contralateral side of experimental rats (Hazer Rosberg et al., 2024).

### 3.3 ATF3 immunoreactivity in the sciatic nerve

**FIGURE 4:**
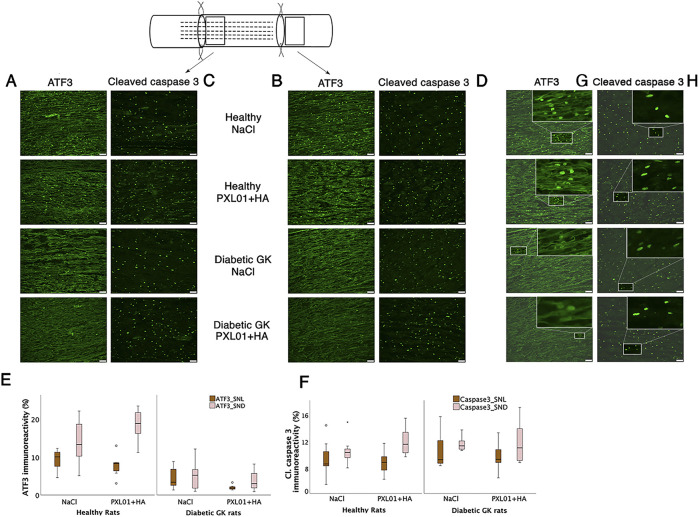
Activated (ATF3 stained) **(A, B)** and apoptotic (cleaved caspase 3-stained) Schwann cells **(C, D)** at the proximal side of lesion (SNL) and at the distal nerve end (SND) in autograft reconstructed sciatic nerve 8 days after the application of different treatment agents in healthy and diabetic GK rats. Scale bar = 50 μm. Box plots show the percentage of ATF3-stained **(E)** and cleaved caspase 3-stained Schwann cells **(F)** in the operated sciatic nerve. The groups are coded according to different treatment agents: sodium chloride treated group, NaCl; and PXL01 with sodium hyaluronate treated group, PXL01+HA. The magnification inserts showing ATF3 expressed **(G)** and cleaved caspase 3 **(H)** expressed Schwann cells are presented at the distal nerve end for each experimental group. Box plots indicate the 25th and 75th percentiles (Tukey’s hinges) with the horizontal line in the middle, indicating the median value. Error bars show minimum and maximum values.

Double-staining for ATF3 and S-100 was performed to confirm the localization and characteristics of ATF3-stained activated Schwann cells, which are long, oval cells oriented parallel to the growing axons (Hazer Rosberg et al., 2021; Hazer Rosberg et al., 2024). ATF3 expression differed significantly between treatment groups, both at the proximal side of the lesion and at the distal nerve end (p = 0.001 at the site of the lesion and p < 0.001 at the distal nerve end, KW test; Figure 4; Table 1). In the NaCl-treated groups, healthy rats had a higher number of activated Schwann cells than diabetic GK rats, both at the proximal side of the lesion and at the distal nerve end (p = 0.01 and p = 0.01, respectively, Mann–Whitney U-test). Similarly, in the PXL01-treated groups, healthy rats had a higher number of activated Schwann cells than diabetic GK rats, both at the proximal side of the lesion and at the distal nerve end (p = 0.001 and p < 0.001, respectively; Mann Whitney U-test). However, we did not observe a significant difference between NaCl and PXL01 treatments at the proximal side of the lesion or at the distal nerve end in the healthy rat group or in the diabetic GK rat group (p = 0.07 and p = 0.73, respectively, for diabetic GK rats; p = 0.31 and p = 0.16, respectively, for healthy Wistar rats; Mann–Whitney U-test). There was no significant difference between ATF3 expression at the proximal side of lesion and at the distal nerve end in NaCl-treated groups between the healthy and diabetic GK rats (p = 0.05 for the Wistar-NaCl group; p = 0.86 for the GK-NaCl group; Wilcoxon signed rank test), but in the PXL01 treated groups, the distal nerve end had a higher number of activated Schwann cells than the proximal side of lesion (p = 0.01 for the Wistar-PXL01 group; p = 0.04 for the GK-PXL01 group; Wilcoxon signed rank test). The uninjured contralateral sciatic nerve contained only a few ATF3-stained cells.

### 3.4 Cleaved caspase 3 immunoreactivity in the sciatic nerve

Double-staining for cleaved caspase 3 and S-100 was performed to ensure the localization and characteristics of the ATF3-stained cells as activated Schwann cells (Hazer Rosberg et al., 2021; Hazer Rosberg et al., 2024). There was no significant difference between the treatment groups with respect to the percentage of cleaved caspase 3 immunoreactivity in the operated sciatic nerve, both at the proximal side of the lesion and at the distal nerve end (p = 0.67 and p = 0.42, respectively, KW test; Figure 4; Table 1). The cleaved caspase 3 immunoreactivity at the proximal side of the lesion was similar to the immunoreactivity at the distal nerve end in NaCl-treated groups (p = 0.31 in Wistar-NaCl group; p = 0.31 in the GK-NaCl group; Wilcoxon signed rank test) but was higher at the distal nerve end than the at proximal side of the lesion in the PXL01-treated groups (p = 0.02 in the Wistar-PXL01 group; p = 0.04 in the GK-PXL01 group; Wilcoxon signed rank test). The uninjured contralateral sciatic nerve showed no staining for cleaved caspase 3.

### 3.5 CD68 immunoreactivity in sciatic nerve

CD68 (ED1) is a pan-macrophage marker that stains activated macrophages (Wofford et al., 2022). Double-staining for S-100 and NF was performed to ensure the localization and characteristics of CD68-stained activated macrophages, as described in a previous study (Hazer Rosberg et al., 2024). At both the proximal side of the lesion and the distal nerve end, CD68-stained macrophages were usually round and large, sometimes forming giant cells. The contralateral uninjured nerves showed very little CD68 staining.

**FIGURE 5:**
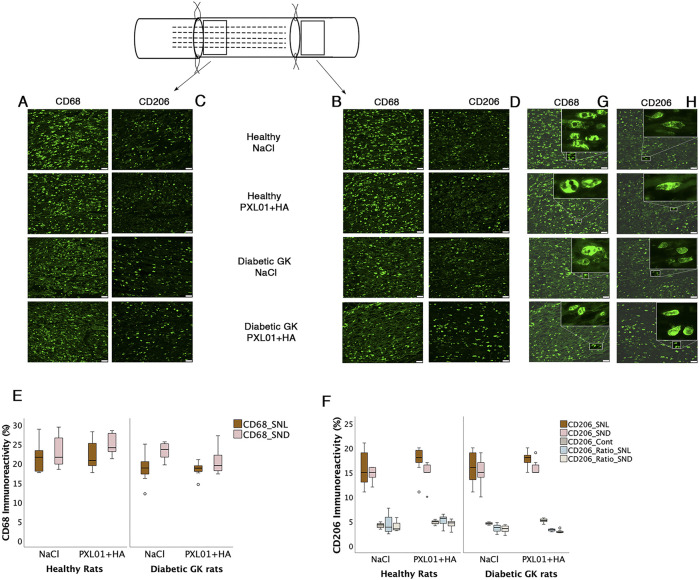
Presence of CD68 and CD206 stained macrophages at the proximal side of lesion **(A, C)** and at the distal nerve end **(B, D)** in the autograft reconstructed sciatic nerve 8 days after applications of different treatment agents in healthy Wistar and in diabetic GK rats are presented. Bar = 50 μm. The magnification inserts showing CD68 expressed **(G)** and CD206 **(H)** expressed macrophages are presented at the distal nerve end for each experimental group. Box plots of percentage of CD68 stained **(E)** and CD206 stained macrophages **(F)** in the operated sciatic nerve (SNL = proximal side of lesion; SND = distal nerve end) and in the contralateral nerve (Cont) are shown. The groups are coded according to different treatment agents: sodium chloride treated group, NaCl; and PXL01 with sodium hyaluronate treated group, PXL01+HA. Box plots indicate the 25th and 75th percentiles (Tukey’s hinges) with the horizontal line in the middle, indicating the median value. Error bars show minimum and maximum values.

There was no significant difference in CD68 immunoreactivity between the proximal side of the lesion and the distal nerve end (p = 0.26 and p = 0.11, respectively, KW test; Figure 5; Table 2). Comparison of the experimental groups did not reveal statistically significant differences. The percentage of CD68 immunoreactivity at the distal nerve end was significantly higher than that at the proximal side of the lesion in the NaCl-treated diabetic GK rat group (p = 0.04, GK-NaCl group; Wilcoxon signed rank test), but there were no differences between the proximal side of the lesion and the distal nerve end in the other groups (p = 0.46, Wistar-NaCl group; p = 0.06, Wistar-PXL01 group; p = 0.12, GK-PXL01 group; Wilcoxon signed rank test). The PXL01-treated healthy Wistar rats had a higher CD68 count at the distal nerve end than the PXL01-treated diabetic GK rats (p = 0.03, Mann–Whitney U test).

**TABLE 2:** Continued data of the immunohistochemical analysis of the regeneration process in the sciatic nerve, using different antibodies, at 8 days in the autograft reconstructed sciatic nerve injury model in healthy Wistar rats and diabetic Goto-Kakizaki (GK) rats after treatment with sodium chloride (NaCl) and PLX01 in sodium hyaluronate (PXL01+HA).

Sciatic nerve		Healthy Wistar rats		Diabetic Goto-kakizaki rats		
		NaCl (n = 7)	PXL01+HA (n = 7)	NaCl (n = 7)	PXL01+HA (n = 7)	p-values
CD68 - experimental side (% total number DAPI positive cells)	Site of lesion	22 (18–25)	21 (19–26)	19 (16–21)	19 (18–20)	0.26
	Distal nerve end	22 (19–28)	24 (23–28)	24 (21–25)	19 (18–25)	0.11
CD206 - experimental side (% total number DAPI positive cells)	Site of lesion	15 (13–20)	18 (16–20)	16 (13–20)	18 (16–19)	0.73
	Distal nerve end	15 (14–16)	15 (15–17)	15 (13–17)	15 (15–17)	0.72
CD206 - contralateral side (% total area)		4.3 (3.6–4.6)^b^	5.0 (4.3–5.2)	4.6 (4.4–4.8)^b^	5.2 (4.7–5.5)^a^	**0.01**
CD206 ratio - experimental/contralateral	Site of lesion	3.8 (2.6–7.5)	5.6 (4.6–6)	3.7 (2.8–4.3)	3.3 (2.9–3.5)	0.08
	Distal nerve end	3.5 (3.2–5.7)^b^	4.7 (3.8–5.1)^a^ ^,^ ^b^	3.5 (2.6–4.0)	2.8 (2.7–3.1)	**0.02**
DAPI positive cells - experimental side (no/mm^2^)	Site of lesion	3,045 (2,824–3,279)^a^ ^,^ ^b^	3,037 (2,853–3,076)^a^ ^,^ ^b^	2015 (1705–2,658)	2,143 (1873–2,440)	**0.003**
	Distal nerve end	2,958 (2,622–3,381)	2,871 (2,584–3,121)	2,670 (2,556–2,801)	2,524 (2,404–2,778)	0.06
Blood glucose (mmol/L)		5.3 (3.7–5.6)^a^ ^,^ ^b^	3.9 (3.5–4.8)^a^ ^,^ ^b^	12.4 (11.0–13.2)	12.0 (10.6–12.4)	**<0.001**

CD68-stained and CD206-stained macrophages are expressed as percentages of the total number of DAPI-positive cells. DAPI, cell count, and blood glucose levels are presented. Data are presented as median (25th −75th percentiles). P-values were based on the Kruskal–Wallis test. Significant p values (p < 0.05) are indicated in bold. NaCl, sodium chloride treatment group; PXL01+HA, PXL01 in the sodium hyaluronate carrier treatment group. DAPI, 4′,6-diamino-2-phenylindole.

^a^ indicates a statistical difference compared to the GK-NaCl group.

^b^ indicates a statistical difference compared to the GK-PXL01 group.

### 3.6 CD206 immunoreactivity in the sciatic nerve

The percentages of CD206 stained macrophages at the proximal side of the lesion and at the distal nerve end in the operated sciatic nerve were similar in all treatment groups, with no statistically significant differences (p = 0.73, proximal side of the lesion; p = 0.72 distal nerve end, KW test; Table 2). In all treatment groups, the CD206 count in the operated sciatic nerve both at the proximal side of the lesion and at the distal nerve end was higher than the cell count in the contralateral unoperated sciatic nerve (p = 0.02 for all treatment groups; Wilcoxon signed rank test). The percentage of CD206-stained macrophages in the contralateral nerve and the CD206 ratio (experimental/control) at the distal nerve end were significantly different in experimental groups with a complex pattern of statistical difference (p = 0.01 and p = 0.02, KW test; Figure 5; Table 2). In the unoperated contralateral sciatic nerve, PXL01 treatment of both healthy and diabetic rats caused an increase in the CD206 count (p = 0.02 for the diabetic rats and p = 0.03 for the healthy Wistar rats; Mann–Whitney U-test). The ratio of the CD206 count at the proximal side of the lesion was higher than that at the distal nerve end in PXL01-treated healthy Wistar rats (p = 0.01, Wilcoxon signed rank test). Healthy Wistar rats had a higher ratio of CD206 count both at the proximal side of the lesion and at the distal nerve end than did diabetic GK rats treated with PXL01 (p = 0.01 and p = 0.007, respectively; Mann–Whitney U-test).

Comparison of the CD206 count at the proximal side of the lesion and at the distal nerve end revealed no difference for all treatment groups except for PXL01-treated Wistar rats in which the CD206 count was higher in the proximal side of lesion compared to the distal nerve end (p = 0.35 for the Wistar-NaCl, p = 0.02 for the Wistar-PXL01; p = 0.49 for the GK-NaCl, p = 0.21 for the GK-PXL01 group; Wilcoxon signed rank test).

### 3.7 DAPI-stained cells in the sciatic nerve and blood glucose

The analyses of DAPI-stained cells, indicating the total cell number of cells and expressed as number per square millimeter, revealed a statistically significant difference at the proximal side of the lesion (p = 0.003 at the proximal side of the lesion, p = 0.06 at the distal nerve end, KW test; Table 2). Healthy Wistar rats had a higher total cell count in both the NaCl-treated and PXL01-treated groups than diabetic GK rats at the proximal side of the lesion (p = 0.02 for NaCl treatment and p < 0.001 for PXL01 treatment; Mann–Whitney U-test). There was no difference in the total DAPI cell count between the proximal side of the lesion and the distal nerve end in all treatment groups.

Blood glucose levels were significantly higher in diabetic GK rats than in healthy Wistar rats, without any significant difference between the treatment groups (p < 0.001, KW test; Table 2). Diabetic GK rats had higher blood glucose levels in both the NaCl-treated and PXL01-treated groups than healthy Wistar rats (p < 0.001 for both NaCl and PXL01 treatments, Mann–Whitney U-test).

### 3.8 Immunoreactivity in the DRG

**FIGURE 6:**
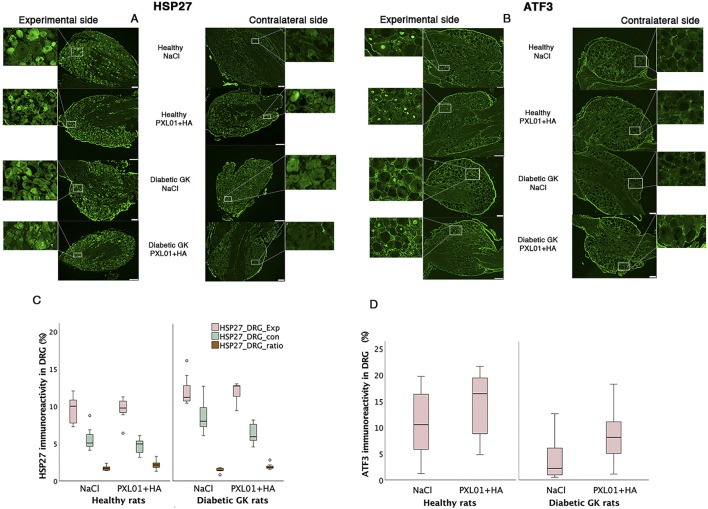
HSP27 immunoreactivity **(A)** and ATF3-stained sensory neurons **(B)** in the DRGs in operated (experimental) and contralateral side 8 days after application of different treatment agents in healthy and diabetic GK rats. Scale bar = 100 μm. Magnified inserts indicating the HSP27 expression in the sensory neuron **(A)** in the experimental side and in the contralateral side for each treatment modality and health status of the rats. Magnified inserts indicating the ATF3 expression in the sensory neuron **(B)** in the experimental side and in the contralateral side for each treatment modality and health status of the rats. Box plots show HSP27 immunoreactivity **(C)** and percentage of ATF3-stained sensory neurons **(D)** in DRGs. The groups are coded according to different treatment agents: sodium chloride treated group, NaCl; and PXL01 with sodium hyaluronate treated group, PXL01+HA. Exp: experimental side, con: contralateral side. Box plots indicate the 25th and 75th percentiles (Tukey’s hinges) with the horizontal line in the middle indicating the median value. Error bars show minimum and maximum values.

HSP27 expression in the experimental DRGs was significantly different between the experimental groups (p = 0.02, KW test; Table 3; Figure 6). In the PXL01-treated groups, diabetic GK rats had higher HSP27 immunoreactivity in the experimental DRGs than healthy rats (p = 0.03, Mann–Whitney U test). For all treatment groups, the expression of HSP27 in the DRG on the operated side was significantly higher than that on the contralateral side (p = 0.03 for GK-NaCl, p = 0.02 for the other treatment groups; Wilcoxon signed rank test). HSP27 expression in the contralateral DRG differed between treatment groups (p = 0.005, KW test), such that diabetic GK rats had higher HSP27 immunoreactivity in the contralateral DRG than both NaCl-treated (p = 0.01, Mann–Whitney U-test) and PXL01-treated (p = 0.04, Mann–Whitney U-test) healthy rats. Additionally, in diabetic rats, comparison between treatment modalities revealed that PXL01 treatment resulted in a decrease in HSP27 immunoreactivity in the contralateral DRG (p = 0.04, Mann–Whitney U-test), but at the same time, an increase in the ratio of HSP27 in DRGs (p = 0.03, Mann–Whitney U-test). The HSP27 ratio (experimental/control) did not differ between the treatment groups (p = 0.07, KW test; Table 3).

**TABLE 3:** The percentage of heat shock protein 27 (HSP27) immunoreactivity and the percentage of activating transcription factor 3 (ATF3)-stained sensory neurons in dorsal root ganglion (DRG) in the uninjured contralateral and in the operated sides together with the ratio of HSP27 expression in DRG (experimental site to the contralateral uninjured site) after autograft reconstruction of the sciatic nerve in healthy and diabetic GK rats 8 days after surgery and treatment with sodium chloride (NaCl) and PLX01+sodium hyaluronate as a carrier.

Dorsal root ganglia (L4 - L5 DRG)	Healthy Wistar rats		Diabetic Goto-kakizaki rats		
	NaCl (n = 7)	PXL01+HA (n = 7)	NaCl (n = 7)	PXL01+HA (n = 7)	p-values
HSP27 - contralateral side (%)	5.0 (4.4–6.8)^a^	4.9 (3.4–5.4)^b^	8.0 (6.8–10.2)^b^	5.9 (5–7.9)	**0.005**
HSP27 - experimental side (%)	10.0 (7.6–11.5)^b^	9.8 (8.8–11.0)^a^ ^,^ ^b^	11.2 (10.4–14.1)	12.7 (10.2–12.8)	**0.02**
HSP27 ratio - experimental/contralateral	1.7 (1.4–1.9)	2.1 (1.6–2.6)	1.4 (1.3–1.7)	1.8 (1.6–2.1)	0.07
ATF3 stained sensory neurons - experimental side (%)	10.5 (5.2–16.4)^a^	16.4 (8.1–20.5)^a^	2.2 (0.8–8.4)	8.1 (3.5–13.9)	**0.04**

Data are presented as median (25th −75th percentiles). NaCl, sodium chloride treatment group; PXL01+HA, PXL01 in the sodium hyaluronate carrier treatment group. P-values were based on the Kruskal–Wallis test. Significant p values (p < 0.05) are indicated in bold. DRG, dorsal root ganglion.

^a^ indicates a statistical difference compared to the GK-NaCl group.

^b^ indicates a statistical difference compared to the GK-PXL01 group.

The ATF3-stained sensory neurons in the operated DRGs were found to be different between the experimental groups (p = 0.04, KW test; Table 3; Figure 6), being higher in healthy rats than in diabetic GK rats treated with NaCl (p = 0.04, Mann–Whitney U-test). There was no difference between PXL01 and NaCl treatment in different health statuses of the rats (p = 0.30, healthy Wistar rats, Mann–Whitney U-test; p = 0.15, diabetic GK rats, Mann–Whitney U-test). The contralateral DRG contained only a few ATF3-stained sensory neurons.

### 3.9 Two-way ANOVA

Two-way ANOVA was used to compare the effects on the length of axonal outgrowth, activity of Schwann cells, HSP27 expression, presence of macrophages in the sciatic nerve, and ATF3 immunoreactivity in the DRG and the local treatment of PLX01 and health status of rats (healthy vs diabetic) (Table 4).

**TABLE 4:** Summary of the ANOVAs in the sciatic nerve or in dorsal root ganglia (DRG) after autograft reconstruction of the sciatic nerve in healthy and diabetic GK rats 8 days after surgery and treatment with sodium chloride (NaCl) and PLX01+sodium hyaluronate as a carrier.

Effect	MS	DF	F	*p-value*	Effect size
Axonal outgrowth					
Diabetes	8903811.832	1	4.803	0.038*	0.167
Treatment	12083373.342	1	6.519	0.017*	0.214
Diabetes × Treatment	4520290.181	1	2.439	0.131	0.092
ATF3 in nerve - site of lesion					
Diabetes	188.760	1	28.639	0.000*	0.544
Treatment	29.623	1	4.494	0.045*	0.158
Diabetes × Treatment	2.184	1	0.331	0.570	0.014
ATF3 in nerve - distal nerve end					
Diabetes	976.333	1	47.643	0.000*	0.665
Treatment	18.959	1	0.925	0.346	0.037
Diabetes × Treatment	55.329	1	2.700	0.113	0.101
HSP27 in nerve - contralateral					
Diabetes	10.504	1	6.595	0.017*	0.216
Treatment	0.000	1	0.000	0.996	0.000
Diabetes × Treatment	2.189	1	2.002	0.170	0.077
HSP27 ratio in nerve - site of lesion					
Diabetes	1.793	1	2,972	0.098	0.110
Treatment	0.385	1	0.638	0.432	0.026
Diabetes × Treatment	0.043	1	0.072	0.791	0.003
HSP27 ratio in nerve - distal nerve end					
Diabetes	0.907	1	1.497	0.233	0.059
Treatment	0.351	1	0.597	0.454	0.024
Diabetes × Treatment	1.414	1	2.334	0.140	0.089
CD68 in nerve - site of lesion					
Diabetes	76.825	1	5.720	0.025*	0.192
Treatment	0.268	1	0.020	0.889	0.001
Diabetes × Treatment	2.063	1	0.154	0.699	0.006
CD68 in nerve - distal nerve end					
Diabetes	24.388	1	2.869	0.103	0.107
Treatment	0.799	1	0.670	0.798	0.003
Diabetes × Treatment	34.788	1	2.902	0.101	0.108
CD206 in nerve - contralateral					
Diabetes	1.003	1	4.943	0.036*	0.171
Treatment	2.703	1	12.318	0.001*	0.375
Diabetes × Treatment	0.003	1	0.016	0.901	0.001
CD206 ratio in nerve - site of lesion					
Diabetes	13.970	1	7.988	0.090	0.250
Treatment	0.138	1	0.079	0.781	0.003
Diabetes × Treatment	1.846	1	1.056	0.314	0.042
CD206 ratio in nerve - distal nerve end					
Diabetes	7.697	1	10.238	0.004*	0.299
Treatment	0.010	1	0.013	0.910	0.001
Diabetes × Treatment	1.481	1	1.970	0.000*	0.076
ATF3 in DRG - experimental					
Diabetes	263.529	1	7.182	0.013*	0.230
Treatment	103.758	1	2.828	0.106	0.105
Diabetes × Treatment	1.603	1	0.044	0.836	0.002
HSP27 in DRG - contralateral					
Diabetes	39.961	1	14.724	0.001*	0.380
Treatment	19.506	1	7.187	0.013*	0.230
Diabetes × Treatment	3.016	1	1.111	0.302	0.044
HSP27 ratio in DRG					
Diabetes	0.465	1	2.209	0.150	0.084
Treatment	1.458	1	6.922	0.015*	0.224
Diabetes × Treatment	0.001	1	0.006	0.938	0.000

ANOVA, results showing the treatment and batch effects on axonal outgrowth, Schwann cell activation in the sciatic nerve and DRG, HSP27 in the sciatic nerve and DRG, and CD68 and CD206 stained macrophages in the sciatic nerves (*p < 0.05). The mean square (MS), degrees of freedom (DF), F-test value (F), p-values, and effect sizes are presented.

Diabetes = diabetic Goto-Kakizaki rats compared with healthy Wistar rats. Treatment = treatment with PLX01 in a sodium hyaluronate carrier, compared with placebo = NaCl; saline.

Effect sizes are classified as:

0.01–0.059 = small.

0.06–0.139 = medium.

0.14 and above = large (Cohen, 1988).

#### 3.9.1 Axonal outgrowth and activated and neuroprotective events in the sciatic nerve

**FIGURE 7:**
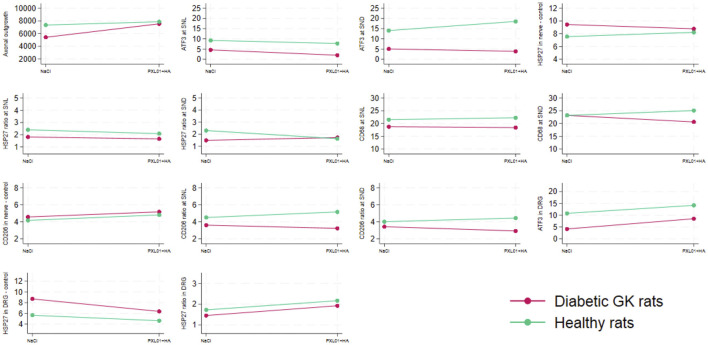
Interaction plots indicating the effects on the variables listed in Table 4 by health status (i.e., diabetic Goto-Kakizaki rats and healthy Wistar rats) and treatment (NaCl, saline; placebo and treatment with PLX01 in sodium hyaluronate carrier; PLX01+HA). For the location of the analyses, see Figure 1. SNL = at site of lesion. SND = at distal nerve end.

Concerning axonal outgrowth, significant effects of health status and treatment were observed, both with a large size effect (Table 4; Figure 7); i.e., the significant effect of PLX01 treatment (p = 0.017) on axonal outgrowth showed a large effect size (0.214), indicating that the treatment improved axonal outgrowth in both healthy and diabetic GK rats. However, the observed lack of a significant interaction between treatment and health (diabetes) status (p = 0.131; Table 4) supported the notion that the treatment effect was not significantly different between the two health status groups. Significant effects were also found concerning the activation of Schwann cells (i.e., ATF3 expression) at the proximal side of the lesion, both for health status and treatment (both large effect sizes), and at the distal nerve end only for health status (large effect size). In addition, a significant effect of health status was found only for HSP27 expression in the contralateral control nerves (large effect size). The ratio of HSP27 expression (experimental side/control side) in the nerve was calculated, and neither the ratio at the lesion site nor that at the distal nerve end was significant (Table 4; Figure 7).

#### 3.9.2 Macrophages in sciatic nerve

The presence of CD68 in the sciatic nerve was significantly affected by health status (large effect size) but not by treatment at the site of the lesion; no significant effects of health status or treatment were observed at the distal nerve end. In the CD206-stained control sciatic nerves, health status as well as treatment had significant effects (both large effect size; Table 4; Figure 7). The CD206 ratio at the site of the lesion showed no significant effect, whereas the CD206 ratio at the distal nerve end was significantly affected by health status and health status × treatment (large and medium effect sizes, respectively).

#### 3.9.3 Activation and neuroprotection in DRGs

For activation in the DRG, indicated by ATF3 staining in sensory neurons, a significant effect was found for health status (large effect size) but not for treatment. HSP27 staining in the control DRG, which is indicative of neuroprotection, showed significant effects on health status and treatment (both large effect sizes). Furthermore, HSP27 staining of the DRG, expressed as a ratio (i.e., experimental side/control side), showed significant effects of treatment (large effect size) (Table 4; Figure 7).

The significant effects, based on the two-way ANOVA, can be summarized as follows:

a. Health status and treatment were significant factors for axonal outgrowth, ATF3 expression at the proximal site of the lesion, CD206 expression in the control nerve, and HSP27 expression in the control DRG.
b. A significant effect was found only for health status on ATF3 expression at the distal nerve end, HSP27 in the control nerve, CD68 at the proximal site of the lesion, CD206 ratio at the distal nerve end, and ATF3 in the DRG.
c. Concerning only treatment, a significant effect was found for HSP27 ratio in the DRG.
d. No significant effects were observed in HSP27 ratios at the proximal site of the lesion and at the distal nerve ends, CD68 ratio at the distal nerve end, or CD206 ratio at the site of the lesion.
e. No significant interactions were found between health status and treatment for most variables, except for the CD206 ratio at the distal nerve end.

## 4 Discussion

In the present study, we investigated the effect of local application of the lactoferrin-derived peptide PXL01 in a sodium hyaluronate carrier on autograft-reconstructed nerve injuries in healthy and diabetic GK rats at a short-term follow-up, using a previously used experimental model (Danielsen et al., 1993; Kerns et al., 1993). We found that local application of PXL01 and the diabetic health status of rats in the present autograft model influenced axonal outgrowth and the neuroprotective response. This neuroprotection may include both “protection-survival of the neurons and the Schwann cells” as well as “activation of a regrowth program” that may inhibit the risk for neuronal cell death in DRG and HSP27 which may be a decisive factor in diabetes (Korngut et al., 2012; Pourhamidi et al., 2014; Shi et al., 2001; Tandrup et al., 2000; Vestergaard et al., 1997; West et al., 2013). The length of axonal outgrowth was shorter in diabetic GK rats at 8 days in accordance with previous data obtained using a similar surgical protocol (Stenberg et al., 2016). The present findings with a longer axonal outgrowth by PXL01 treatment are contrary to the observation in healthy Wistar rats 6 days after nerve transection injury with direct nerve repair, in which PXL01 decreased the number of CD68- but not CD206-expressing macrophages, indicating an effect of the PXL01 treatment on inflammation but not on nerve regeneration (Hazer Rosberg et al., 2024). Our recent findings using direct and immediate nerve repair (Hazer Rosberg et al., 2024) were also contrary to those of a clinical study in which signs of improved axonal outgrowth after a repaired digital nerve injury were observed (Wiig et al., 2014). The treatment with PLX01 showed a statistically significant large effect size (0.214) on axonal outgrowth in both healthy and diabetic rats, where the lack of a significant interaction between treatment and diabetes status further supported that the treatment effect was not different between the two health status groups.

PXL01 reduces the expression of PAI-1, an inhibitor of the plasminogen activator system that regulates fibrin clearance and adhesion formation (Nilsson et al., 2009). The plasminogen activator system may enhance nerve regeneration (Siconolfi and Seeds, 2001) and is found to be a neuroprotective agent in the central nervous system against hypoxia (Yepes, 2015). It can be postulated that PXL01, by reducing PAI-1 and therefore enhancing the expression of the plasminogen-activating system, can enhance axonal outgrowth more in a hypoxic environment, such as in the early stages of nerve reconstruction with an autograft, compared to the primary nerve repair model (Hazer Rosberg et al., 2024). A further assumption is that local application of PXL01 may generally be more effective in a hypoxic environment, thereby benefiting nerve regeneration in other models, such as nerve conduits used to bridge nerve defects. By the factorial ANOVA, the ratio of the expression of HSP27 in the sensory neurons in DRG, but not in the sciatic nerve (i.e., present in both axons and Schwann cells), were higher after local application of PXL01 around the autograft, which indicates a neuroprotective effect on the sensory neurons after application of the drug (Dahlin, 2023; Read and Gorman, 2009). However, there was a contralateral effect on the uninjured side, with lower expression of HSP27 in sensory neurons in the DRG, which may indicate a systemic effect of PXL01 despite the lack of any contralateral effect on the sciatic nerve. Systemic effects by release of pharmacological substances may be difficult to judge as in other model systems of wounds. PXL01 is probably rapidly released from the vehicle at the site of application in accordance what has been shown *in vitro* (Nilsson et al., 2009; Pfalzgraff et al., 2018), which may lead to a systemic effect although of lesser extent. In addition, effects of hyaluronic acid, the presently used vehicle, have mainly been observed *in vitro* (Bajic et al., 2024) and with modest effects after nerve repair or reconstruction using a guide with hyaluronic acid bridging a nerve gap (Ozgenel, 2003; Wang et al., 1998), but without any effect after direct nerve repair after injury in a similar time perspective as the present study (Bajic et al., 2024; Hazer Rosberg et al., 2024; Ozgenel, 2003; Wang et al., 1998). With this background, and with the 3R principle in mind, we decided to use only NaCl as placebo and PXL01 as the experimental treatment.

In our previous study, using a primary nerve repair model in healthy rats to which PXL01 was locally applied, inflammation was decreased, as observed by a lower number of CD68-stained pan-macrophages, but there was no change in the CD206-stained pro-healing macrophages (Hazer Rosberg et al., 2024). Interestingly, in the present nerve reconstruction model using autografts, in which microcirculation initially is not present, PXL01 had no effect on the presence of macrophages, except that diabetic GK rats had a lower percentage of CD68-stained macrophages, and no direct effect was observed on CD206-stained macrophages at the lesion site. Additionally, the two-way ANOVA revealed that diabetes decreased the ratio of CD206 count at the distal nerve end (Table 4). In our previous study on primary nerve repair in healthy rats, we did not observe any effect of PXL01 treatment on CD206 count. These alterations could be explained by diabetes affecting the regeneration cascade after nerve injury (Msheik et al., 2022; Sango et al., 2017).

Local application of PXL01 decreased the number of ATF3-stained Schwann cells at the site of the lesion but not at the distal nerve end, which may be a combined effect of the treatment (potential effect of CD68-stained macrophages) and the environment, which was initially without vascularization (see below). Both diabetic health status and local PXL01 treatment seemed to decrease the number of ATF3-stained Schwann cells, especially at the site of the lesion. Surprisingly, neither treatment with PXL01 nor health status affected the presence of cleaved caspase 3-stained Schwann cells in the factorial ANOVA analysis. However, PXL01 treatment showed an increased number of apoptotic and activated Schwann cells at the distal nerve end compared to that at the site of the lesion in the reconstructed sciatic nerves; a biological phenomenon that may be attributed to the impact of the axon-Schwann cell interaction with downregulation in Schwann cells at the site of lesion. Overall, the data show that both health status and local treatment with PXL01 affect the distal nerve in the present nerve reconstruction model using nerve autografts, where Wallerian degeneration regulates Schwann cell activity in a complex manner.

Intraneural microcirculation may be the key factor explaining the difference in the effect of PXL01 between the nerve repair and nerve reconstruction models. In autografts, cellular responses, such as Wallerian degeneration, mimic the cellular reaction that occurs at the distal nerve end just distal to the suture site. These grafts undergo remodeling in the following weeks, including the invasion of macrophages, proliferation of donor graft Schwann cells, degeneration of axons and myelin debris within the graft, and migration of Schwann cells between the graft and repaired nerve ends (Pan et al., 2020). Schwann cells that are already present in the grafted nerve segment are dependent on diffusion initially and later, on revascularization. Vascularization in an autograft is first restored by host vessels that grow into the autograft going from the proximal and distal nerve ends (Pan et al., 2020; Saffari et al., 2020), especially from the proximal side of the lesion (Saffari et al., 2020).

The present study included diabetic GK rats to study the effects of PXL01 in a diabetic environment. Diabetes, with a high systemic blood glucose level, leads to increased oxidative stress and an increase in free radicals (Albers and Pop-Busui, 2014), resulting in neuropathy by four proposed mechanisms (Brownlee, 2005). Schwann cells are thought to lose their capacity to provide energy to axons through a mechanism that triggers the influx of extracellular calcium ions inside the axon, which disturbs axonal mitochondrial trafficking, resulting in insufficient energy generation and mitochondrial apoptosis (Fernyhough, 2015). This pathophysiological mechanism is especially important in autograft reconstruction models of nerve injury explaining the lower percentage of activated Schwann cells at both sites in diabetic rats, independent of treatment modality. Our experimental model, with a shorter follow-up time, showed a decreased length of axonal outgrowth, which contradicts previous findings, and a lower number of ATF3-stained Schwann cells, which agrees with previous findings (Stenberg et al., 2016). The time required to prepare the environment of the autograft in diabetes may take some time, which may be related to restoration of microcirculation (Stenberg et al., 2016). In addition to the changes in the operated sciatic nerve after autograft reconstruction with the local application of PXL01, there were also alterations in the distant response to injury and reconstruction in the DRGs. In the present study, we observed that diabetes caused higher HSP27 immunoreactivity in the contralateral DRGs but decreased the activated sensory neuron count. We have previously shown that different surgical repair models cause an increase in HSP27 expression, both in the uninjured contralateral nerve and in the contralateral DRGs (Stenberg et al., 2021), indicating that diabetes has somehow a neuroprotective effect on the contralateral sciatic nerve (i.e., in the axons and Schwann cells) and on sensory neurons in the DRG, possibly as a response to a low-grade neuropathy, where HSP27 is a decisive protective factor (Korngut et al., 2012; Pourhamidi et al., 2014). Taken together, these data indicate that diabetes affects the uninjured sensory neurons, and Schwann cells and has a negative effect on axonal outgrowth and on the numbers of activated Schwann cells and CD68 pan-macrophages after nerve injury and nerve reconstruction with autografts.

Upon acute peripheral nerve injury, RAGE (receptors for advanced glycation end products [AGEs]) becomes expressed in activated Schwann cells and infiltrating mononuclear phagocytes (Sorci et al., 2013). In a nerve crush model, proinflammatory type M1 macrophages are significantly higher in diabetic wild-type mice than in non-diabetic mice, but RAGE-deficient diabetic mice exhibit a decrease in M1 macrophages and an increase in anti-inflammatory type M2 macrophages relative to those in diabetic wild-type mice, which have led to a hypothesis that an impaired axonal regeneration in diabetic mice is at least partly attributable to a RAGE-dependent alteration of macrophage polarization (Juranek et al., 2013). Our findings contradict the literature in the sense that diabetic GK rats had a lower CD68 pan-macrophage count at the site of the lesion (i.e., in the autograft, which is initially an avascular segment of the nerve), but not at the distal nerve end. The decrease in the number of CD68 pan-macrophages, together with the decreased ratio of CD206 pro-healing macrophages, may contribute to the shorter axonal outgrowth detected in diabetic GK rats, together with the lower number of ATF3-stained Schwann cells (Stenberg and Dahlin, 2014; Stenberg et al., 2016). The higher CD206 pro-healing macrophage count in the contralateral nerve after autograft reconstruction and local treatment with PXL01 is a novel finding that could be explained by the well-known systemic response to nerve injury (Verge et al., 2020).

## 5 Conclusion

Local application of the lactoferrin-derived peptide PXL01 in a sodium hyaluronate vehicle in a nerve reconstruction model with autografts after nerve injury improves the length of axonal outgrowth and induces a better neuroprotective response (i.e., HSP27 expression) in the DRG, but not in the autograft, in healthy and diabetic GK rats at a short (8-day) follow-up. Diabetic health status induces a neuroprotective response in the sciatic nerve and in the DRG on the uninjured side and impairs axonal outgrowth and activation of Schwann cells, together with some effects on the pan- and pro-healing macrophages, in the autografts and at the distal nerve end, as well as in sensory neurons in the DRG after nerve reconstruction at the present short-term follow-up. These data imply that the local application of PXL01 may be more effective in a hypoxic environment for nerve regeneration.

## Data Availability

The datasets presented in this study can be found in online repositories. The names of the repository/repositories and accession number(s) can be found in the article/Supplementary Material.
