# Supplementary material for: Lactoferrin-derived peptide PXL01 impacts nerve regeneration after sciatic nerve reconstruction in healthy and diabetic rats
Source: Front Cell Dev Biol. 2025 Apr 7;13:1565285. doi: 10.3389/fcell.2025.1565285 (PMC12009942; doi:10.3389/fcell.2025.1565285)
Supplement: Supplementary file 1 [file Table1.pdf]

Supplementary Material

Supplementary Table 1.

		Primary antibody	Secondary antibody
**Sciatic nerve**	Neurofilament	Monoclonal mouse anti-human neurofilament (1:80; Dako, Glostrup, Denmark)	Alexa Fluor 594-goat anti-mouse IgG (1:500, Invitrogen, Molecular Probes, Eugene, Oregon, USA)
	Heat Shock Protein 27 (HSP27)	Polyclonal rabbit anti-HSP-27 (1:200, ADI-SPA-803, Enzo Life Sciences, Farmingdale, NY, USA)	Alexa Fluor 488-goat anti-rabbit IgG for the HSP27 (1:250, Invitrogen, Molecular Probes, Eugene, Oregon, USA)
	Activating transcription factor 3 (ATF3)	Monoclonal mouse anti-ATF3 (1:200, Sc 81189, Santa Cruz Biotechnology, Dallas, TX, USA)	Alexa Fluor 488-goat anti-mouse IgG (1:500, Invitrogen, Molecular Probes, Eugene, Oregon, USA)
	Cleaved caspase 3	Monoclonal rabbit anti-cleaved caspase 3 (1:200, Cell signalling Technology, Denver, MA, USA)	Alexa Fluor 488-goat anti-rabbit IgG (1:500, Invitrogen, Molecular Probes, Eugene, Oregon, USA)
	CD68	Monoclonal mouse anti-rat CD68 (ED1) (MCA 341R, 1:400, BioRad, Dallas, TX, USA)	Alexa Fluor 488-goat anti-mouse IgG (1:500, Invitrogen, Molecular Probes, Eugene, Oregon, USA)<br><br>Alexa Fluor 594-goat anti-mouse IgG (1:500, Invitrogen, Molecular Probes, Eugene, Oregon, USA)
	CD206	Monoclonal rabbit anti-mannose receptor (CD206) antibody (1:400, ab300621, Abcam, Cambridge, UK)	Alexa Fluor 488-goat anti-rabbit IgG (1:500, Invitrogen, Molecular Probes, Eugene, Oregon, USA)
	S-100	Monoclonal mouse IgG anti S-100 α/β chain (1:300, sc-58839, Santa Cruz Biotechnology Inc., Dallas, TX, USA)	Alexa fluor 594 goat anti-mouse IgG (Invitrogen, Life Technologies Corporation, Carlsbad, CA, USA)

**Dorsal root ganglia (DRG)**	Heat Shock Protein 27 (HSP27)	Polyclonal rabbit anti-HSP-27 (1:200, ADI-SPA-803, Enzo Life Sciences, Farming- dale, NY, USA)	Alexa Fluor 488-goat anti-rabbit IgG (1:250, Invitrogen, Molecular Probes, Eugene, Oregon, USA)
	Activating transcription factor 3 (ATF3)	Monoclonal mouse anti-ATF3 (1:200, Santa Cruz Biotechnology, Dallas, TX, USA)	Alexa Fluor 488-goat anti-mouse IgG (1:500, Invitrogen, Molecular Probes, Eugene, Oregon, USA)
