# Supplementary material for: Lactoferrin-derived peptide PXL01 impacts nerve regeneration after sciatic nerve reconstruction in healthy and diabetic rats
Source: Front Cell Dev Biol. 2025 Apr 7;13:1565285. doi: 10.3389/fcell.2025.1565285 (PMC12009942; doi:10.3389/fcell.2025.1565285)
Supplement: Supplementary file 2 [file DataSheet1.pdf]

Rat number	Rat type GK=1 W=2
1	1
2	1
3	1
4	1
12	1
13	1
14	1
5	1
6	1
7	1
8	1
9	1
10	1
11	1
1	2
2	2
8	2
9	2
12	2
13	2
14	2
3	2
4	2
5	2
6	2
7	2
10	2
11	2

Rat&treatment;GKNaCL=1,GKPXL=2,WNaCL=3,WPXL=4	Neurofilament
1.00	1954
1.00	2894
1.00	6780
1.00	8106
1.00	7442
1.00	6599
1.00	4166
2.00	7534
2.00	7998
2.00	8525
2.00	7242
2.00	6529
2.00	7838
2.00	7096
3.00	7612
3.00	6134
3.00	8233
3.00	6956
3.00	7707
3.00	6944
3.00	7874
4.00	6839
4.00	8768
4.00	8926
4.00	7914
4.00	7714
4.00	6739
4.00	8132

Treatment NaCl=1,PXL=2	HSP27_SNL	HSP27_SND	HSP27_Contr	HSP27_Ratio_SNL
1.00	16.10	12.00	11.37	1.42
1.00	24.10	14.50	10.47	2.30
1.00	15.70	10.40	9.57	1.64
1.00	15.90	18.00	9.01	1.76
1.00	11.80	12.20	9.16	1.29
1.00	17.70	11.10	6.92	2.56
1.00	16.90	16.60	9.63	1.75
2.00	13.90	19.90	9.53	1.46
2.00	12.80	21.50	9.03	1.42
2.00	14.90	15.70	8.15	1.83
2.00	17.70	11.60	8.61	2.06
2.00	9.20	9.00	8.44	1.09
2.00	18.40	8.10	9.73	1.89
2.00	15.00	19.10	7.93	1.89
1.00	8.00	10.50	9.63	.83
1.00	11.60	12.10	7.04	1.65
1.00	22.54	13.52	8.10	2.78
1.00	17.44	24.43	6.32	2.76
1.00	18.90	13.00	9.02	2.10
1.00	23.67	21.94	5.02	4.72
1.00	15.29	15.26	7.70	1.99
2.00	9.60	5.60	9.27	1.32
2.00	23.91	11.38	7.41	3.23
2.00	11.21	12.86	9.25	1.21
2.00	20.34	12.40	7.79	2.61
2.00	11.80	17.12	9.87	1.50
2.00	15.58	12.50	7.34	2.12
2.00	17.48	13.30	6.64	2.63

HSP27_Ratio_SND	ATF3_SNL	ATF3_SND	Caspase3_SN	Caspase3_SN	CD68_SNL
1.06	7.32	6.06	9.00	10.74	12.12
1.67	3.02	2.30	8.45	11.80	16.10
1.09	1.32	5.22	9.33	11.07	18.38
2.00	6.27	12.14	8.60	10.70	18.80
1.33	8.86	.97	13.84	13.78	20.80
1.60	3.39	7.41	10.54	12.47	25.00
1.72	2.05	1.30	15.76	11.40	20.11
2.09	1.60	3.00	9.01	11.10	19.88
2.38	3.30	8.16	10.20	9.41	18.77
1.93	2.32	4.87	13.35	14.31	14.53
1.35	1.84	.92	9.40	13.63	20.99
1.07	1.50	1.50	6.62	8.91	18.27
.83	2.03	6.53	11.48	17.14	18.90
2.41	1.33	2.26	8.80	9.02	17.54
1.09	10.06	15.63	8.38	14.94	17.99
1.72	10.78	22.16	11.70	11.36	17.95
1.67	6.10	5.10	8.70	10.41	21.60
3.87	9.03	10.00	8.46	9.59	25.00
1.44	4.60	10.50	5.63	8.11	28.87
4.37	11.80	21.70	14.46	10.50	17.62
1.98	12.30	13.30	9.40	9.70	21.67
.77	8.28	23.48	8.95	11.64	18.84
1.54	8.58	22.50	6.41	10.84	20.77
1.39	7.00	17.91	9.64	15.52	26.00
1.59	8.50	21.00	11.78	12.18	17.63
2.43	13.00	18.90	7.04	14.67	24.42
1.70	3.07	14.60	8.51	9.90	20.00
2.00	5.75	11.20	9.93	9.77	28.21

CD68_SND	HSP27_DRG_	HSP27_DRG_Exp	HSP27_DRG_ratio
19.63	10.40	12.68	.82
25.00	11.00	6.07	1.80
23.63	14.12	10.20	1.40
25.20	10.40	7.70	1.35
25.60	11.20	8.00	1.40
22.75	11.40	6.84	1.70
20.56	16.09	9.42	1.70
18.37	12.40	8.15	1.52
19.55	13.00	7.30	1.77
17.87	12.71	4.55	2.79
27.17	12.80	5.92	2.15
17.27	12.74	7.86	1.62
24.73	10.25	5.77	1.77
19.44	9.40	5.08	1.84
18.44	10.00	6.85	1.45
18.73	7.93	4.10	1.93
21.60	7.57	4.45	1.70
20.91	7.25	5.08	1.42
28.26	12.04	8.74	1.37
29.42	11.47	4.83	2.37
24.92	10.18	5.54	1.83
28.51	11.25	5.24	2.14
23.32	6.39	4.95	1.30
27.25	8.85	3.38	2.61
21.22	10.99	5.44	2.02
22.87	9.76	6.06	1.61
24.06	10.39	3.16	3.28
28.29	9.45	4.27	2.21
